# Aptamer-Based Sandwich Assay Formats for Detection and Discrimination of Human High- and Low-Molecular-Weight uPA for Cancer Prognosis and Diagnosis

**DOI:** 10.3390/cancers14215222

**Published:** 2022-10-25

**Authors:** Nico Dreymann, Wiebke Sabrowski, Jennifer Danso, Marcus M. Menger

**Affiliations:** 1 Fraunhofer Institute for Cell Therapy and Immunology (IZI), Branch Bioanalytics and Bioprocesses (IZI-BB), D-14476 Potsdam, Germany; 2 Institute for Biochemistry and Biology, University of Potsdam, D-14476 Potsdam, Germany; 3 Institute of Chemistry and Biochemistry, Freie Universität Berlin, D-14195 Berlin, Germany; 4 Berliner Hochschule für Technik (BHT), D-13353 Berlin, Germany

**Keywords:** ALISA, aptamer, biomarker, cancer prognosis, early stage cancer detection, ELONA, sandwich assay, uPA, urokinase

## Abstract

**Simple Summary:**

Urokinase-type plasminogen activator (urokinase, uPA) is a widely discussed biomarker for cancer prognosis and diagnosis. The gold standard for the determination of protein biomarkers in physiological samples is the enzyme-linked immunosorbent assay (ELISA). Here, antibodies are used to detect the specific protein. In our study, recently published urokinase aptamers were tested for their use in a sandwich assay format as alternative specific recognition elements. Different aptamer combinations were used for the detection of uPA in a sandwich-assay format and a combination of aptamers and antibodies additionally allowed the differentiation of human high and low molecular weight- (HMW- and LMW-) uPA. Hence, uPA aptamers offer a valuable alternative as specific recognition elements for analytical purposes. Since aptamers are easy to synthesize and modify, they can be used as a cost-effective alternative in sandwich assay formats for the detection of uPA in physiological samples.

**Abstract:**

Urokinase-type plasminogen activator (urokinase, uPA) is a frequently discussed biomarker for prognosis, diagnosis, and recurrence of cancer. In a previous study, we developed ssDNA aptamers that bind to different forms of human urokinase, which are therefore assumed to have different binding regions. In this study, we demonstrate the development of aptamer-based sandwich assays that use different combinations of these aptamers to detect high molecular weight- (HMW-) uPA in a micro titer plate format. By combining aptamers and antibodies, it was possible to distinguish between HMW-uPA and low molecular weight- (LMW-) uPA. For the best performing aptamer combination, we calculated the limit of detection (LOD) and limit of quantification (LOQ) in spiked buffer and urine samples with an LOD up to 50 ng/mL and 138 ng/mL, respectively. To show the specificity and sequence dependence of the reporter aptamer uPAapt−02−FR, we have identified key nucleotides within the sequence that are important for specific folding and binding to uPA using a fluorescent dye-linked aptamer assay (FLAA). Since uPA is a much-discussed marker for prognosis and diagnosis in various types of cancers, these aptamers and their use in a micro titer plate assay format represent a novel, promising tool for the detection of uPA and for possible diagnostic applications.

## 1. Introduction

Early cancer detection and therapy are one of the most important tasks of today’s medicine as the number of cancer incidences is increasing and, according to the World Health Organization (WHO), will rise rapidly in the next years. Many studies have focused on the molecular level of tumor development, using aberrant expression of various proteins as potential targets for cancer therapy or biomarkers for cancer diagnosis. Compared to traditional histological characterization of tumors, they enable impressive progress as a non-invasive alternative for cancer prognosis and diagnosis [1].

Urokinase-type plasminogen activator (urokinase, uPA) is one of the many proteins involved in various processes necessary for cancer progression and is therefore widely discussed as a potential therapeutic target and a prognostic and diagnostic marker for human malignancies [2,3]. Urokinase is a serine protease consisting of 411 amino acid residues. Through cleavage of the K158-I159 bridge by several proteases, uPA is activated to the two-chain high molecular weight uPA (HMW-uPA). It is composed of the N-terminal A-chain containing the growth factor domain (GFD) and the kringle domain (KD), and the C-terminal B-chain containing the catalytically active serine protease domain (SPD), both linked by a disulfide bond. Further cleavage between Lys135 and Lys136 of HMW-uPA results in two fragments—the amino terminal fragment (ATF) with the GFD and KD, and the C-terminal catalytic domain that forms the catalytically active low molecular weight uPA (LMW-uPA) [3].

While the ATF (especially the GFD) is responsible for binding to the uPA receptor (uPAR), the catalytic domain of uPA is responsible for the conversion of plasminogen into plasmin, which promotes proteolysis of fibrinogen into fibrin and degradation of the extracellular matrix [3]. This is crucial for the early steps of tumor progression by promoting the expansion of tumor mass and release of tumor growth factors, as well as inducing tumor cell proliferation, migration, invasion, and angiogenesis [4].

Elevated expression of uPA is shown in many different types of cancer and mainly correlates with poor prognosis [3,4,5]. For example, high levels of uPA in breast cancer tissue extracts showed clinical significance in primary breast cancer patients and can be used as an independent prognostic biomarker of overall and relapse-free survival [6,7]. Therefore, the Tumor Marker Guideline of the American Society of Clinical Oncology (ASCO) recommended uPA biomarker testing by ELISA to assess the risk of recurrence in node negative breast cancer patients and to decide individually if adjuvant chemotherapy is beneficial after surgery [8]. In a recent study, it was shown that uPA can serve alongside other biomarkers as a prognostic marker in the blood of patients with metastatic breast cancer [9].

High levels of uPA were found in plasma of patients with prostate cancer, which is correlated with increased aggressiveness, postoperative progression, and metastasis [10,11]. Urokinase was suggested by Yang et al. [12] to be used as an independent prognostic factor for colorectal cancer patients’ survival and metastasis. Herszéyi et al. [13] have proved that circulating uPA can be used as better prognostic markers than the commonly used colorectal cancer markers CEA and CA 19-9 as increased uPA levels could be detected in the blood of colorectal cancer patients. Increased uPA concentrations could also be found in the plasma of bladder cancer patients compared to healthy controls [14]. Recent studies showed that it could be used for personalized clinical decision-making. Concentrations of uPA showed the potential as a predictor for aggressiveness and worse survival outcomes in patients with urothelial carcinoma of the bladder after radical cystectomy and it was also associated with recurrence-free and cancer-specific survival [15].

Besides elevated uPA levels in different tumor tissues and blood, a higher uPA concentration was found in the urine of bladder cancer patients [16]. Shariat et al. [17] have shown that urinary uPA could be used as a diagnostic biomarker to improve the ability to predict bladder cancer besides cytology and other urinary biomarkers.

The enzyme-linked immunosorbent assay (ELISA) is the basic method for detection and determination of uPA concentrations, as it is still the gold standard for the detection of proteins in physiological samples. Commercially available kits using different antibodies in a sandwich-assay format allow the detection and determination of uPA concentrations in different samples. Besides antibodies, aptamers offer the possibility as alternative molecular recognition elements, as they recognize their targets with high specificity and affinity comparable to antibodies. The single-stranded (ss) nucleic acid (ssDNA or RNA) molecules have the advantages that they are easy and cheap to produce, can easily be modified, are smaller in size, and are more chemically stable [18]. Therefore, aptamers are a promising tool for the detection of target molecules [19] and for in vitro detection strategies of cancer (e.g., cancer biomarkers) [20]. Hence, previously selected uPA aptamers were used to test whether they can be used as a substitute for antibodies in a sandwich assay format for analytical purpose, as they target different uPA epitopes [21].

**Figure 1:**
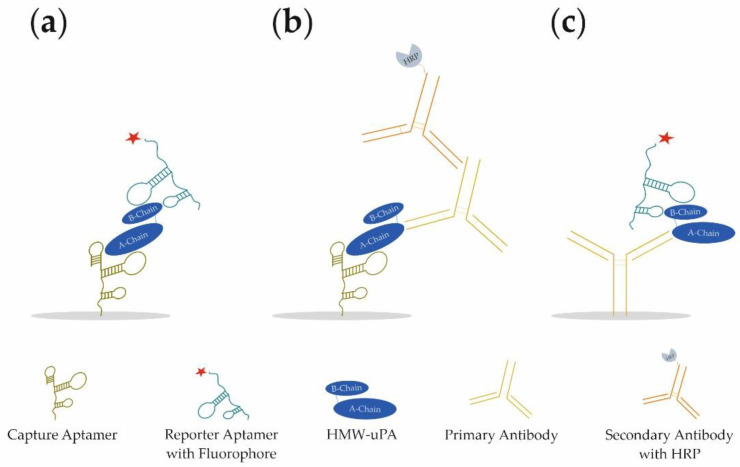
Schematic illustration of the three different sandwich assay formats developed for the detection of different forms of human urokinase. (**a**) Aptamer–Target–Aptamer, (**b**) Aptamer–Target–Antibody, and (**c**) Antibody–Target–Aptamer.

In our study, we present the development of a novel aptamer-based sandwich assay (aptamer–target–aptamer; Figure 1a) using different aptamers for the detection of HMW-uPA. By combining different aptamers and antibodies, it was possible to detect both HMW-uPA and LMW-uPA in different sandwich assay formats (aptamer–target–antibody or antibody–target–aptamer; Figure 1b,c, respectively). For the best aptamer combination of the aptamer-based sandwich assay, the limit of detection (LOD) and limit of quantification (LOQ) was determined in buffer conditions, as well as spiked urine samples. For the best reporter aptamer uPAapt−02−FR, we have identified key nucleotides within the aptamer sequence that are important for the specific folding and binding to uPA demonstrating sequence dependence for specific detection of uPA. Since uPA is a much-discussed marker for prognosis and diagnosis in various types of cancer, the use of the aptamers in a sandwich assay format represents a novel, promising, and cost-effective tool for the detection of uPA and possibly for prognostic and diagnostic applications.

## 2. Materials and Methods

### 2.1. Urokinase and Aptamer Preperation

Native HMW-uPA isolated from human urine was purchased from ProSpec-Tany TechnoGene Ltd. (Ness-Ziona, Israel). LMW-uPA was prepared as previously described [21]. For the fluorescent-dye linked aptamer assays (FLAA), HMW-uPA was biotinylated using EZ-Link™ Sulfo-NHS-SS-Biotin (Thermo Fisher Scientific Inc., Waltham, MA, USA) according to the manufacturer’s instructions. All chemicals were purchased from Carl Roth GmbH + Co. KG, Karlsruhe, Germany. Aptamers were obtained from three different providers (biomers.net GmbH, Ulm, Germany; IBA Lifesciences GmbH, Göttingen, Germany; Integrated DNA Technologies, Inc., Coralville, IL, USA).

All uPA aptamers [21] were tested in micro titer plate assay formats. For this purpose, 5′-biotinylated uPA aptamers were used as capture aptamers and 5′-Cyanine(Cy)-5-labled aptamers were used as reporter aptamers. Prior to use of aptamers in the respective assay formats, aptamers were refolded. Therefore, aptamers were diluted to a specific concentration in BPs-T (50 mM Bis-Tris/HCl pH 6.5, 110 mM NaCl, 5 mM MgCl_2_, 1 mM CaCl_2_, 1 mM KCl_2_, 0.05% *v/v* Tween® 20) and incubated it at 92 °C for 3 min and then slowly (for about 30 min) cooled to 25 °C. All solutions were prepared in a total volume of 100 µL if not indicated otherwise.

### 2.2. Aptamer-Based Sandwich Assay (Sandwich-ALISA)

For the development of an aptamer-linked immobilized sorbent assay (ALISA) in a sandwich format, 5′-biotinylated aptamers were used as capture aptamers on black Pierce™ streptavidin-coated high-capacity 96-well micro titer plates (Thermo Fisher Scientific Inc., Waltham, MA, USA). All incubation and washing steps were carried out at 23 °C and 300 rpm in a thermoshaker. Before immobilization of capture aptamers and between each incubation step, wells were washed thrice with 300 µL BPs-T for 90 s. For the immobilization of capture aptamers, 25 pmol of each refolded 5′-biotinylated aptamer in BPs-T was added to each well and incubated for 15 min. To block remaining biotin binding sites, wells were blocked subsequently with 5000 pmol biotin in BPs-T for 15 min. For binding of the two different uPA-forms, 40 pmol of HMW-uPA or LMW-uPA– either in BPs-T or human urine—was added to different wells and incubated for 30 min. Refolded 5′-Cy-5-labled aptamers were used as reporter aptamers for detection of captured uPA. Here, 32 pmol of refolded reporter aptamer in BPs-T was added to each well and incubated for 30 min. Subsequently, wells were washed thrice with BPs-T. Fluorescence was measured either by EnVision^®^ 2105 multimode plate reader (excitation 620 nm, emission 685 nm, top mirror Cy5; PerkinElmer Inc., Waltham, MA, USA) or by multimode microplate reader Mithras^2^ LB 943 (excitation 610 nm, emission 665 nm; Berthold Technologies GmbH & Co. KG, Bad Wildbad, Germany). The limit of detection and limit of quantification were calculated from three independent experiments, with each three technical replicates using a linear calibration curve. LOD and LOQ were calculated by the following formula: 3 × σ/k and 10 × σ/k, respectively, where σ is the standard deviation of y-intercepts of regression lines and k is the slope of the calibration curve [22].

### 2.3. Fluorescent Dye-Linked Aptamer Assay (FLAA)

To identify relevant binding nucleotides, a fluorescence-based binding assay with the nucleotide-exchanged sequence aptamers was carried out on a black Pierce™ streptavidin-coated high-capacity 96-well micro titer plate (Thermo Fisher Scientific Inc., Waltham, MA, USA) as previously described [21]. All incubation and washing steps were carried out at 23 °C and 300 rpm in a thermoshaker. Before and between each incubation step, wells were washed thrice with 300 µL BPs-T for 90 s. Here, biotinylated HMW-uPA (35 pmol) was immobilized in 100 µL BPs-T for 60 min in different wells. Subsequently, wells were incubated with 50 µM biotin in 200 µL BPs-T for 30 min to block remaining biotin-binding residues. Biotin-blocked wells without uPA were used as a no target control (NTC). Before application, all nucleotide-exchanged sequences were refolded as previously described and 32 pmol of refolded aptamers in BPs T were added to each well and incubated for 60 min at 23 °C. After washing for 90 s at 300 rpm, 100 µL of a 1:200 dilution of Invitrogen™ Quant-iT™ OLIGREEN™ (Fisher Scientific GmbH, Schwerte, Germany) in BPs-T was added to each well. Following an incubation step of 12 min, fluorescence (excitation 485 nm, emission 535 nm) was measured by a multimode microplate reader Mithras^2^ LB 943 Monochromator Multimode Reader (Berthold Technologies GmbH & Co. KG, Bad Wildbad, Germany). Binding of the nucleotide-exchanged sequence aptamers was tested using different micro titer plates. Therefore, uPAapt−02−FR was carried along in each experiment as the positive control. All new sequence aptamers as well as the positive control were tested as duplicates.

For the determination of the maximum binding capacity of capture aptamers immobilized on Pierce™ streptavidin-coated high-capacity 96-well micro titer plates, a modified version of the FLAA was used. Here, a 5′-biotinylated aptamer was incubated at different concentrations (0 pmol–100 pmol) in 100 µL BPs-T for 30 min in different wells. Subsequently, wells were washed thrice, incubated with 100 µL of a 1:200 dilution of Invitrogen™ Quant-iT™ OLIGREEN™ and measured as described above.

### 2.4. Aptamer–Antibody-Based Sandwich Assay

For the aptamer–antibody-based sandwich assay, 5′-biotinylated aptamers were used as capture aptamers on clear Pierce™ streptavidin-coated high-capacity plates (Thermo Fisher Scientific Inc., Waltham, MA, USA). Antibodies, binding either only HMW-uPA (binding to chain A of uPA) or binding HMW- and LMW-uPA (binding to chain B of uPA), were used for detection of different captured uPA-forms. All incubation and washing steps were carried out at 23 °C and 300 rpm in a thermoshaker. Before immobilization of capture aptamers and between each incubation step, wells were washed thrice with 300 µL BPs-T for 90 s. For the immobilization of capture aptamers, 25 pmol of each refolded 5′-biotinylated aptamer in BPs-T was added to each well and incubated for 60 min. To block remained biotin binding sites, wells were blocked with 5000 pmol biotin in 100 µL BPs-T for 60 min. For binding of the two different uPA-forms, 40 pmol of either HMW-uPA or LMW-uPA in BPs-T was added to different wells and incubated for 60 min. For the detection, two different antibodies were used. Anti-PLAU antibody (anti-PLAU antibody (AA16-115), ABIN562262, antibody-online GmbH, Aachen, Germany), which binds to Chain A of uPA, was used for detection of HMW-uPA (Ab Chain A). Anti-uPA antibody [U-16] (Anti-uPA antibody [U-16] ab131433, abcam, Cambridge, UK), which binds to Chain B of uPA, was used for detection of HMW- and LMW-uPA (Ab Chain B). Antibodies were diluted at a ratio of 1:4000 in Roti^®^Block (Carl Roth GmbH + Co. KG, Karlsruhe, Germany) and 100 µL of either Ab Chain A or Ab Chain B dilution was added to different wells and incubated for 30 min. A secondary polyclonal sheep anti-mouse IgG antibody labeled with horseradish peroxidase (A6782, Sigma-Aldrich, St. Louis, MO, USA) was used for detection of the primary antibodies. For this purpose, the secondary antibody was diluted at a ratio of 1:3000 in Roti^®^Block and 100 µL of the dilution was added to each well and incubated for another 30 min. Afterwards, each well was washed thrice with BPs-T and 100 µL of 1-Step™ Ultra TMB-ELISA Substrate Solution (Thermo Fisher Scientific Inc., Waltham, MA, USA) was added to each well. The reaction was stopped using 50 µL of 2 M H_2_SO_4_. Subsequently, the absorbance at 450 nm was measured by multimode microplate reader Mithras^2^ LB 943.

### 2.5. Antibody–Aptamer-Based Sandwich Assay

For the antibody-aptamer based sandwich assay, different antibodies (binding either only HMW-uPA or binding HMW- and LMW-uPA) were used for capturing the different uPA forms on a black 96-well polystyrene micro titer plate (Fluotrac™ 600 high binding, Greiner Bio-One International GmbH, Frickenhausen, Germany). For detection of different captured uPA forms, 5′-Cy5-labled aptamers were used. All incubation and washing steps were carried out at 23 °C at 300 rpm in a thermoshaker. For immobilization, an excess of antibodies was used to achieve maximum coating of the wells. As the manufacturer specifies a maximum coating of 600 ng protein per cm^2^, 800 ng of either anti PLAU antibody (Ab Chain A) or Anti-uPA antibody [U-16] (Ab Chain B) for binding of HMW- and LMW-uPA in 100 µL 1xPBS were incubated for 3 h. Before and after immobilization of antibodies, wells were washed thrice with 1xPBS for 90 s. To block remained binding sites, wells were blocked with blocking solution (1% *v/v* BSA, 10% *v/v* saccharose in 1xPBS) for another 3 h. Subsequently, wells were washed thrice with BPs-T for 90 s. Then, 40 pmol of the different uPA-forms in BPs-T were added to different wells and incubated for 60 min. After incubation, wells were washed thrice with BPs-T for 90 s. For the detection of the different uPA forms, 5′-Cy5-labled aptamers were used. Therefore, 32 pmol of refolded 5′-Cy5-aptamers in BPs-T was added to each well and incubated for 30 min. Afterwards, wells were washed thrice with BPs-T for 90 s and fluorescence was measured by EnVision^®^ 2105 multimode plate reader (excitation 620 nm, emission 685 nm, top mirror Cy5; PerkinElmer^®^ Inc., Waltham, MA, USA).

## 3. Results

### 3.1. Aptamer-Based Sandwich Assay System for Detection of uPA (Sandwich-ALISA)

#### 3.1.1. Design of the Aptamer-Based Sandwich Assay

**Figure 2:**
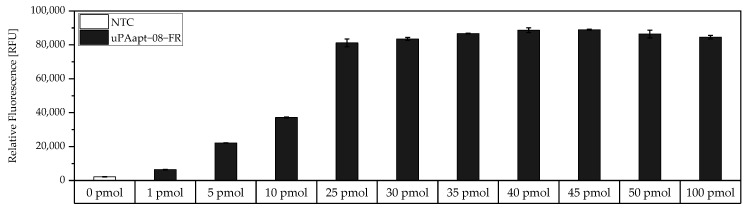
Binding of different amounts of 5′-biotinylated uPAapt−08−FR to the wells of the Pierce™ streptavidin coated high-capacity 96-well micro titer plate by a modified FLAA. Different amounts of uPAapt−08−FR were incubated in different concentrations (0 pmol–100 pmol in 100 µL BPs-T) to the wells to determine the maximum binding capacity for the smallest aptamer. After washing of the wells, followed by incubation with Invitrogen™ Quant-iT™ OLIGREEN™, a fluorescence signal of the immobilized aptamer could be measured. The relative fluorescence unit [RFU] for each sample is shown as the mean value of technical replicates and was measured using the multimode microplate reader Mithras^2^ LB 943. Error bars represent the range of measured values. NTC = No Target Control (0 pmol). Number of records n = 2.

Before testing different aptamer combinations for the aptamer-based sandwich assay, several considerations were made regarding the use of the concentrations of the capture aptamer, the target, and the reporter aptamer, and their respective ratios. To achieve a maximum immobilization of the capture aptamer, a modified FLAA was performed to obtain the maximum binding capacity of an aptamer to the streptavidin-coated wells. For this test, the smallest aptamer, uPAapt−08−FR, was used, as it was assumed that larger aptamers will also achieve the maximum coating of the wells at the same concentration. Results showed that no considerable increase of fluorescence signal was observed above 25 pmol aptamer (Figure 2).

Therefore, 25 pmol capture aptamer were used for the micro titer plate assays to achieve an approximate maximum immobilization of capture aptamers. To add an excess of target, 40 pmol was added in each assay. However, if we assume that the complete 25 pmol of capture aptamers are immobilized and we assume a 1:1 binding model, theoretically, a maximum of 25 pmol target can be captured. In order to detect the entire amount of target, 32 pmol of reporter aptamer were used to also have an excess of the reporter aptamer. Consequently, a ratio of 1:1.6:1.3 (capture aptamer:target:reporter aptamer) was used in the following aptamer-based sandwich assay.

#### 3.1.2. Combination of uPA-Aptamers Enabled Detection of HMW-uPA

**Figure 3:**
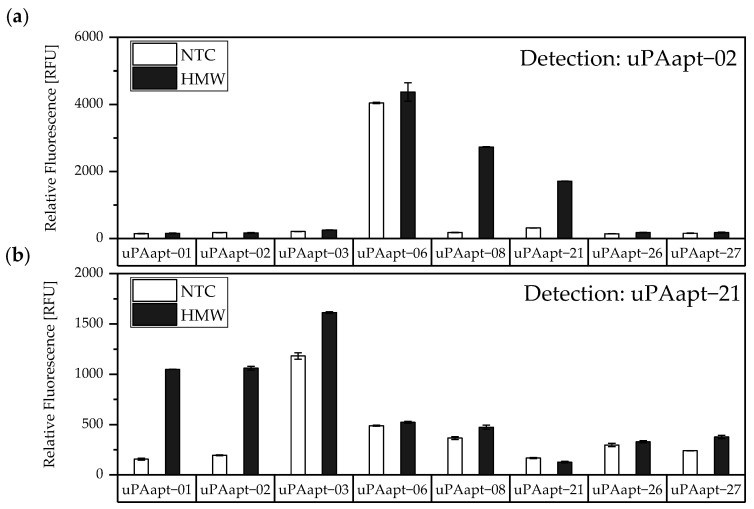
Screening for possible aptamer combinations for detection of HMW-uPA. All aptamers were tested for their use as capture aptamers when (**a**) uPAapt−02 or when (**b**) uPAapt−21 is used as the reporter aptamer. The relative fluorescence unit [RFU] for each sample is shown as the mean value of technical replicates and was measured using the multimode microplate reader Mithras^2^ LB 943. Error bars represent the range of measured values. NTC = No Target Control, HMW = HMW-uPA. Number of records *n* = 2.

Different combinations of uPA aptamers were tested for their functionality in an aptamer-based sandwich assay system to detect the different uPA forms. In order to find suitable aptamer pairs for the development of an aptamer-linked immobilized sorbent assay (ALISA), all full-length uPA aptamers (uPAapt−01, uPAapt−02, uPAapt−03, uPAapt−06, uPAapt−08, uPAapt−21, uPAapt−26, and uPAapt−27) were immobilized on a streptavidin coated micro titer plate via 5’-biotin modification to serve as capture aptamers. The full-length aptamers uPAapt−02 and uPAapt−21 were selected as potential 5’-fluorescently labeled reporter aptamers due to their different binding properties [21]. Thus, selected capture and reporter aptamers were combined in a sandwich assay format using constant concentrations of HMW- or LMW-uPA. The results of the screening for possible aptamer combinations for HMW-uPA are shown in Figure 3.

**Figure 4:**
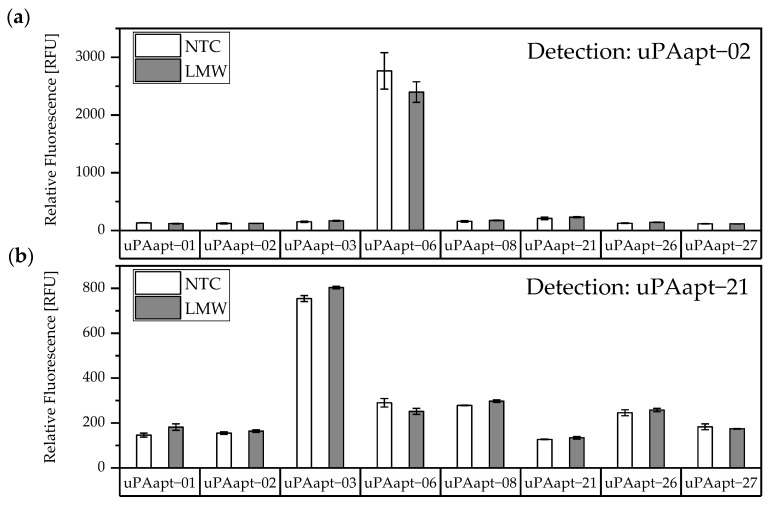
Screening for possible aptamer combinations for detection of LMW-uPA. All aptamers were tested for their use as capture aptamers when (**a**) uPAapt−02 or when (**b**) uPAapt−21 is used as the reporter aptamer. The relative fluorescence unit [RFU] for each sample is shown as the mean value of technical replicates and was measured using the multimode microplate reader Mithras^2^ LB 943. Error bars represent the range of measured values. NTC = No Target Control, HMW = HMW-uPA. Number of records *n* = 2.

The results of the screening showed that different aptamer combinations were able to detect HMW-uPA. This supports the already suspected different binding sites. No signals were detected for LMW-uPA, indicating that these combinations of aptamers are not suitable to detect LMW-uPA and that tested aptamers may not have different binding sites on LMW-uPA (Figure 4).

Additionally, the aptamer combinations of uPAapt−06 and uPAapt−02, as well as uPAapt−03 and uPAapt−21, also showed high signals in the absence of uPA (NTC), which could arise from partial sequence complementarity and does not derive from an aptamer–target interaction. Consequently, these combinations were excluded from the study. Unspecific interactions between other capture and reporter aptamer combinations were not detected. No binding of uPA or the reporter aptamers to the streptavidin coated micro titer plate was detected.

**Figure 5:**
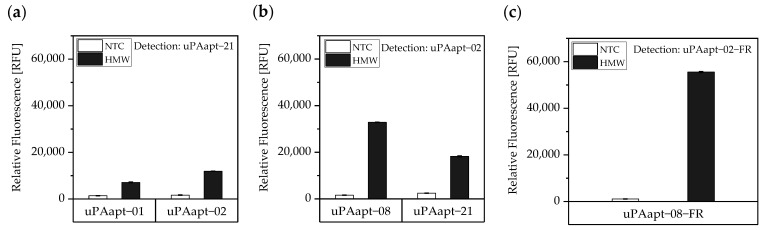
Aptamer-based sandwich assay for detection of HMW-uPA by different combinations of aptamers. HMW-uPA could be detected when (**a**) uPAapt−01 or uPAapt−02 are used as capture aptamers and uPAapt−21 as the reporter aptamer or when (**b**) uPAapt−08 or uPAapt−21 are used as capture aptamers and uPAapt−02 as the reporter aptamer or when (**c**) uPAapt−08−FR is used as a capture aptamer and uPAapt−02−FR as the reporter aptamer. The relative fluorescence unit [RFU] for each sample is given as the mean value of technical replicates and was measured using the EnVision^®^ 2105 multimode plate reader. Error bars represent the range of measured values. NTC = No Target Control, HMW = HMW-uPA. Number of records *n* = 2.

Sandwich assays with suitable aptamer combinations for HMW-uPA were repeated using the EnVision^®^ 2105 multimode plate reader and are shown in Figure 5. Suitable aptamer pairs were uPAapt−21 as a reporter aptamer and uPAapt−01 or uPAapt−02 as capture aptamers (Figure 5a) or uPAapt−02 as a reporter aptamer and uPAapt−08 or uPAapt−21 as capture aptamers (Figure 5b).

As the combination of the full-length aptamers uPAapt−08 (capture aptamer) and uPAapt−02 (reporter aptamer) showed the highest signal for the detection of HMW-uPA, the fully truncated versions (truncated by both primer binding sites) uPAapt−08−FR and uPAapt−02−FR were tested in the aptamer-based sandwich assay. Combination of these two truncated aptamers revealed the highest signal for the detection of HMW-uPA compared to the full-length aptamers and thus serve as best performing aptamer pair for the aptamer-based sandwich assay (Figure 5c).

#### 3.1.3. Aptamer-Based Sandwich Assay Showed a Detection Limit of 50 ng/mL in BPs-T and Still Detects up to 138 ng/mL HMW-uPA in Spiked Urine Samples

**Figure 6:**
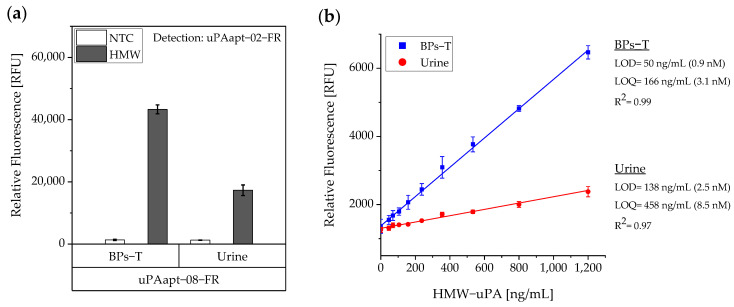
Sensitivity of the aptamer-based sandwich assay for detection of HMW-uPA by uPAapt−08−FR and uPAapt−02−FR in BPs-T and spiked urine samples. (**a**) Comparison of the aptamer-based sandwich assay in BPs-T and spiked urine at a constant concentration of 40 pmol HMW-uPA per well. (**b**) Determination of the sensitivity of the aptamer-based sandwich assay. Calibration curves were obtained by plotting the measured relative fluorescence unit as a function of the HMW-uPA concentration in a standard series. A linear dependence was found between the concentration of HMW-uPA and the measured relative fluorescence units [RFU]. The relative fluorescence is given as the average value of three independent experiments each with three technical replicates and was measured using the EnVision^®^ 2105 multimode plate reader. Error bars represent the standard deviation. NTC = No Target Control, HMW = HMW-uPA. Number of records *n* = 3.

As studies have shown, elevated levels of uPA are found in urine from bladder cancer patients and uPA can serve as a diagnostic marker in human urine. Hence, it was tested whether the aptamer-based sandwich assay also works on human urine samples. For this purpose, human urine was collected on three different days and spiked with a constant concentration of 40 pmol HMW-uPA per well, individually. For comparison, the same was performed in BPs-T. Three independent experiments were performed with three technical replicates each. Results are shown in Figure 6a. Even though the signal was no longer as high as in BPs-T, it was demonstrated that the sandwich assay still showed a high signal compared to the no target control (NTC). To test its sensitivity, the LOD and the LOQ were determined by a linear calibration curve. Here, three independent experiments each with three technical replicates were performed. In each experiment a standard series ranging from 0–1.200 ng/mL HMW-uPA was prepared in BPs-T or human urine and the aptamer-based sandwich assay was performed. The calibration curves were obtained by plotting the measured relative fluorescence units (RFU) as a function of the HMW-uPA concentration. A linear dependence was found between the concentration of HMW-uPA and the measured RFU having a determination coefficient (R^2^) for BPs-T and urine samples with 0.99 and 0.97, respectively. The LOD for the assay in BPs-T was calculated as 50 ng/mL and for spiked human urine as 138 ng/mL. The LOQ for BPs-T was 166 ng/mL and for spiked human urine 458 ng/mL (Figure 6b).

#### 3.1.4. FLAA Experiments Revealed Key Nucleotides That Are Important for the Specific Folding and Binding of uPAapt−02−FR

**Figure 7:**
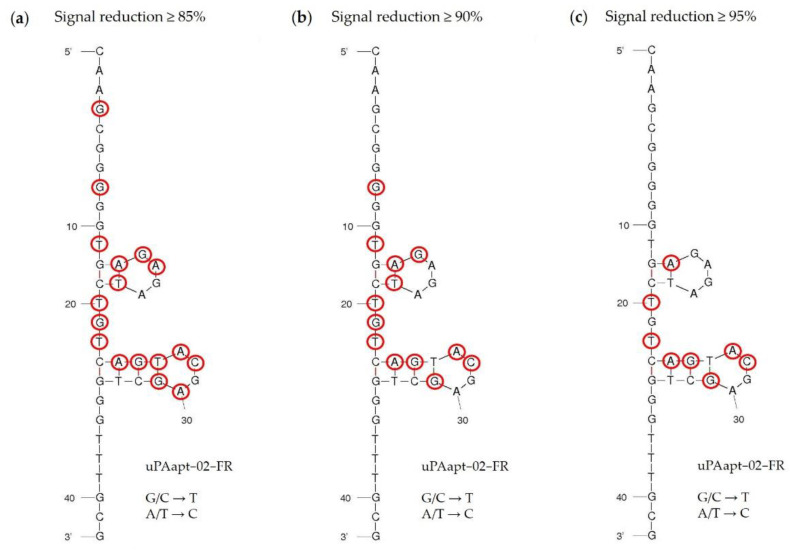
Secondary structure of the reporter aptamer uPAapt−02−FR predicted by mfold. All 42 nucleotides were replaced individually (for G/C a T and for A/T a C) and each sequence was analyzed for binding in FLAA. Exchanged nucleotides for which the resulting aptamer showed a signal reduction of (**a**) ≥85%, (**b**) ≥90%, or (**c**) ≥95% compared to uPAapt−02−FR are circled in red.

As described above, uPAapt−02−FR served as the most promising detection aptamer. The specificity of the reporter aptamer uPAapt−02−FR for human uPA has already been described [21]. To evaluate sequence dependence and to identify key nucleotides within the aptamer sequence, nucleotide exchange sequences were designed. Here, each nucleotide of the sequence was replaced individually (for G/C a T and for A/T a C) and all 42 new sequence aptamers (named Exchange-Sequence 1−42; Ex−S1–Ex−S42) were tested for binding to HMW-uPA by FLAA. The results of the FLAA and a table of all tested sequences are shown in Supplementary Figure S1 and Table S1, respectively. The results of the FLAAs are summarized in Figure 7. Here, the secondary structure of uPAapt−02−FR was visualized using the program mfold [23] which predicted a secondary structure containing two four-base hairpin loop motifs (tetraloops). Exchanged nucleotides for which the resulting aptamer showed a signal reduction of greater than or equal to 85% (Figure 7a), 90% (Figure 7b) or 95% (Figure 7c) compared to uPAapt−02−FR are circled in red. Using FLAA, it was shown that especially the second tetraloop within the sequence predicted by the program mfold appears to be important for the binding affinity to uPA. In conclusion, sequence dependence was shown for the formation of a specific structure that is important for the binding affinity to HMW-uPA.

### 3.2. Combination of Aptamers and Antibodies in a Sandwich-Assay System for Detection and Discrimination of HMW- and LMW-uPA

The aptamer-based sandwich assay was developed as a tool suitable to detect HMW-uPA. However, LMW-uPA could not be detected. Therefore, different combinations of aptamers and antibodies were used to detect both forms of uPA and to distinguish between HMW-uPA and LMW-uPA. For comparability to the aptamer-based sandwich assay, the same concentrations of aptamers and targets were used.

#### 3.2.1. Aptamer–Antibody Sandwich Assay

**Figure 8:**
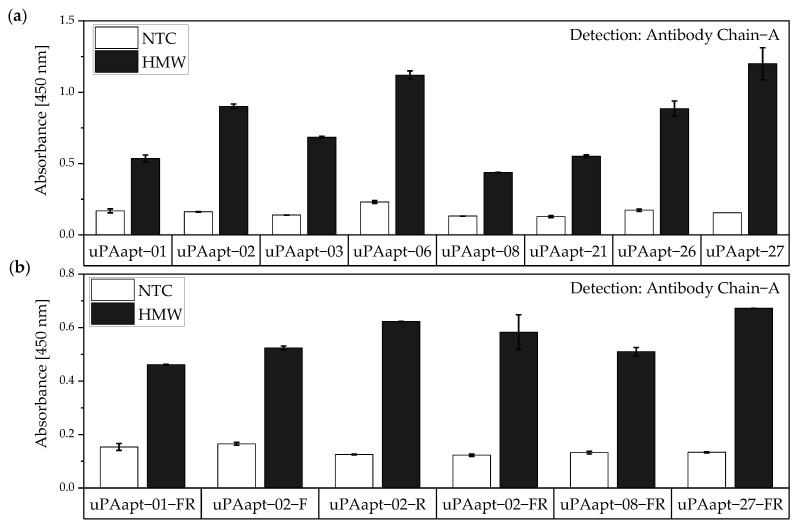
Aptamer–Antibody-based sandwich assay for detection of HMW-uPA by Antibody Chain A (Ab Chain A). HMW-uPA could be detected using (**a**) all uPA aptamers and (**b**) several of the truncated variants as capture aptamers when Ab Chain A is used as a reporter molecule, which only detects the HMW-uPA form due to binding to the A-chain of uPA. The absorbance for each sample is given as the mean value of technical replicates. Error bars represent the range of measured values. Number of records *n* = 2.

For the aptamer–antibody sandwich assay (sandwich ELONA—sandwich enzyme-linked oligonucleotide assay), the aptamers were used as capture molecules and the antibodies as reporter molecules. Here, all uPA aptamers and several truncated variants were used to capture HMW-uPA. Detection of captured HMW-uPA was shown by using the Ab Chain A as a reporter molecule which only binds to HMW-uPA due to binding to the A-chain of human uPA (Figure 8). Results indicate that aptamers and Ab Chain A probably have different binding sites on HMW-uPA and that there is no steric hindrance between the aptamers and the antibody.

**Figure 9:**
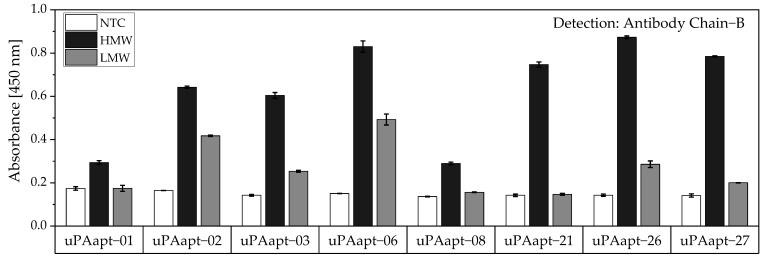
Aptamer–Antibody-based sandwich assay for detection of HMW- and LMW-uPA by Ab Chain B. HMW-uPA could be detected using all uPA aptamers while only uPAapt−02, uPAapt−03, uPAapt−06, uPAapt−26, and uPAapt−27 captured LMW-uPA and could therefore be detected by Ab Chain B, which bind both uPA forms due to binding to the B-Chain of uPA. The absorbance for each sample is given as the mean value of technical replicates. Error bars represent the range of measured values. Number of records *n* = 2.

Detection of captured HMW-uPA could also be shown for the full-length uPA aptamers by using the Ab Chain B which can bind both uPA forms due to binding to the B-chain of uPA. Only the uPA aptamers uPAapt−02, uPAapt−03, uPAapt−06, uPAapt−26, and uPAapt−27 captured LMW-uPA and were detected by Ab Chain B (Figure 9). This indicates that these aptamers bind LMW-uPA and that these aptamers and the antibody may not have the same binding site on LMW-uPA.

**Figure 10:**
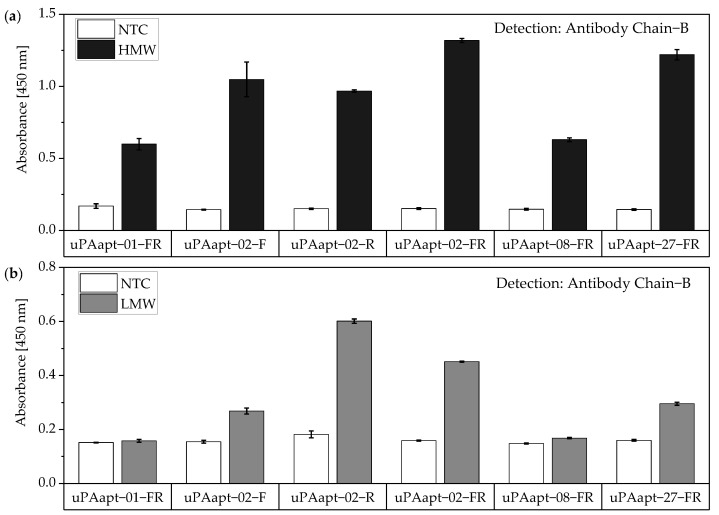
Aptamer–Antibody-based sandwich assay for detection of (**a**) HMW- and (**b**) LMW-uPA by Ab Chain B. HMW-uPA could be detected using all truncated uPA aptamers while only uPAapt−02−F, uPAapt−02−R, uPAapt−02−FR, and uPAapt−27−FR captured LMW-uPA and could therefore be detected by Ab Chain B, which bind both uPA forms due to binding to the B-Chain of uPA. The absorbance for each sample is given as the mean value of technical replicates. Error bars represent the range of measured values. Number of records *n* = 2.

The same assay was performed for the truncated versions of aptamers used as capture molecules. All truncated versions of the aptamers could be used for capturing HMW-uPA if the uPA form is detected by Ab Chain B. Only uPAapt−02−F, uPAapt−02−R, uPAapt−02−FR and uPAapt−27−FR captured LMW-uPA, and therefore could be detected by Ab Chain B (Figure 10).

#### 3.2.2. Antibody–Aptamer Sandwich Assay

**Figure 11:**
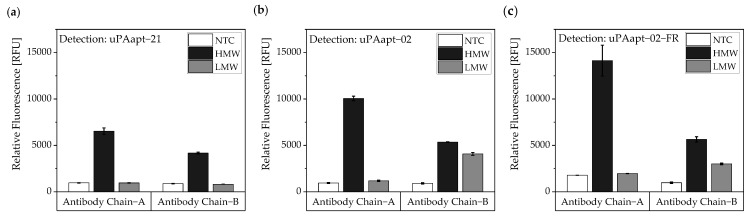
Antibody–Aptamer-based sandwich assay for detection and discrimination of HMW- and LMW-uPA. Antibodies (Ab Chain A or Ab Chain B) were used to capture the different forms of uPA. For detection, (**a**) uPAapt−21 (**b**) uPAapt−02 and (**c**) uPAapt−02−FR were used as reporter molecules. Whereas Ab Chain A can only capture HMW-uPA and therefore uPAapt−02 or the truncated version uPAapt−02−FR can only detect the HMW-uPA form, the Ab Chain B captured the LMW-uPA form and uPAapt−02 or uPAapt−02−FR bind LMW-uPA. The reporter aptamer uPAapt−21 detected only HMW-uPA and showed no binding to LMW-uPA. The relative fluorescence unit [RFU] for each sample is given as the mean value of two technical replicates and was measured using the EnVision^®^ 2105 multimode plate reader. Error bars represent the range of measured values. NTC = No Target Control, HMW = HMW-uPA. Number of records *n* = 2.

For the antibody–aptamer sandwich assay, the antibodies were used as capture molecules and the 5’-fluorescently labeled aptamers as reporter molecules. Ab Chain A captured only HMW-uPA, and therefore, uPAapt−02, or the truncated version uPAapt−02−FR, detected the HMW-uPA form. Using the Ab Chain B, HMW- and LMW-uPA was captured and detected by uPAapt−02 or uPAapt−02−FR. Hence, uPAapt−02 and uPAapt−02−FR maintain binding to LMW-uPA despite binding of the antibody in the region of the serine protease domain. The reporter aptamer uPAapt−21 detected only HMW-uPA and showed no binding to LMW-uPA. (Figure 11).

## 4. Discussion

In this study, we present the use of recently published urokinase aptamers in sandwich assay formats for the detection of the different urokinase forms. Sandwich systems included different formats, such as aptamer–target–aptamer, aptamer–target–antibody, and antibody–target–aptamer. Compared to direct or indirect ELISA or ALISA systems, these sandwich format assays have the advantage of more accurately detecting the target molecule by using two specific detection molecules. Previously, we have shown that aptamers are very likely to target different binding sites, as they can bind either HMW-uPA or HMW- and LMW-uPA [21]. This suggested that the aptamers binding either in the region of the ATF or in the region of the SPD. Because of their different binding sites to uPA, it was possible to detect HMW-uPA in an aptamer-based sandwich assay format using different combinations of aptamers (Figure 5). Here, uPAapt−08−FR used as the capture molecule and uPAapt−02−FR used as the reporter molecule turned out to be the best aptamer combination. Differences in the relative fluorescence signal between the same experiments (Figure 5c and the first two columns of Figure 6a) are probably due to the different labeling efficiencies of different batches of capture and reporter aptamers, which can affect the relative fluorescence signal. Labeling efficiencies with biotin or a fluorescence molecule may vary during chemical synthesis depending on the manufacturer and batch, so aptamers may not be 100% labeled. Variations in the immobilization efficiency caused by differences in biotin labeling efficiency can affect the assay, because different amounts of HMW-uPA may be captured. Furthermore, the fluorescence signal of the reporter aptamer may differ, in case labeling of 5′-Cy5 differs between batches. The quality of the streptavidin-coated plates may also impact the assay.

No tested combination of aptamers was able to detect LMW-uPA in the aptamer-based sandwich assay, indicating that the LMW-uPA binding aptamers tested probably share binding sites in close proximity to each other on LMW-uPA or sterically hinder each other from binding to LMW-uPA. However, the combination of aptamers and antibodies enabled the detection of LMW-uPA—in addition to the detection of the two-chain HMW-uPA form—by some aptamers capable of binding LMW-uPA (Figure 9, Figure 10 and Figure 11). This allows the additional detection of the cleaved serine protease domain and possibly enables the differentiation of the two uPA forms within one sample. Yet, differentiation between the two uPA forms within one sample would have to be validated in further tests by combining different ratios of HMW- and LMW-uPA. In addition, this combination showed that the antibodies and some of the aptamers most likely have different binding sites to HMW- and LMW-uPA.

Specificity of the best performing reporter aptamer, uPAapt−02−FR, has already been shown [21]. In this study, we could also identify key nucleotides within the aptamer sequence that are important for specific folding and binding to uPA, which demonstrates the sequence dependence of the uPA reporter aptamer. Interestingly, for some sequences, the exchange of only one nucleotide was sufficient to reduce binding affinity or impede binding completely. Most of the identified key nucleotides are located near, or are part of, the two four-base hairpin structures in the secondary structure predicted by the program mfold (Figure 7). In particular, signal reduction of equal to or more than 95% compared to uPAapt−02−FR indicates that especially the second hairpin structure is probably very important for binding to uPA (Figure 7c). However, a few nucleotides also appear to be important for binding affinity, which are located outside or between the hairpins formed, as some exchanges showed a signal reduction of at least 85% (Figure 7a,b). These nucleotides may be important for the structural stability of the aptamer. Although these results support the importance of specific structure elements in aptamer–target interactions, further detailed structure analysis, e.g., NMR-spectroscopy or X-ray crystallography, is required in this case to understand the complete role of the identified key nucleotides.

Even though uPA levels are significantly increased in body fluids of cancer patients compared to healthy individuals, uPA concentrations in physiological samples are relatively low. Casella et al. showed that the highest measured concentration of uPA in the urine of bladder cancer patients was 34.1 ng/mL [16] and concentrations of uPA in blood or tissue extracts are far below this level [9,13,14,24]. In a recent study, the optimal cut-off value for serum uPA of patients with metastatic breast cancer was determined as 2.52 ng/mL [9]. Other studies in which uPA was used as a prognostic marker in breast cancer tissue extracts to decide if adjuvant chemotherapy is beneficial in patients with early breast cancer used a cutoff point of 3 ng/mg of protein [7].

For the aptamer-based sandwich assay, a detection limit of 50 ng/mL in buffer conditions was calculated (Figure 6), which is clearly above the concentrations that need to be detected and can be measured by commercially available ELISAs, which are based on antibodies [25]. However, there are several advantages to using aptamers. Aptamers are smaller and are easily produced via chemical synthesis. This is very cost- and time-effective and has less batch-to-batch variation in production [19]. They are also easy to label or modify with different reporter molecules, functional groups, or linkers [26]. Compared to antibodies, aptamers also have the advantage that they do not permanently degrade at elevated temperatures and return to their original conformation when the optimal temperature is reached again. Even if a micro titer plate-based method is normally intended for single use, the capture aptamers could be reused due to their ability to refold after denaturation by heat, high salt solutions, or other chemicals [19].

There are several different strategies to enhance aptamer affinity and specificity during the aptamer selection process and/or after selection which could enhance the overall assay sensitivity and specificity [27]. Several chemical modifications of the bases, sugar moieties, or the phosphate backbone of aptamers after selection could improve their binding affinity [28,29]. Other chemical modifications using synthetic alternatives to natural nucleic acids (DNA or RNA)—so called Xeno Nucleic Acids (XNAs), including, for example SOMAmers (slow off-rate modified aptamers) or Locked Nucleic Acids (LNAs)—could also improve aptamer affinity and thus assay sensitivity [30,31,32]. Using other modifications for immobilization strategies, e.g., an amino group at the 5′- or 3′-end for amine-coupling of the aptamers, or other detection strategies, for example, by the biotin-streptavidin-HRP(Horseradish peroxidase)-system, could also be tested to improve assay sensitivity.

Although there is still some work to be done to improve the sensitivity of the aptamer-based sandwich assay, given these advantages of using aptamers and the possibilities to improve sensitivity, the use of aptamers in micro titer plate-based assays is interesting. As a future attempt, it can also be tested whether a combination of aptamer and antibody has better LOD and LOQ than a combination of aptamers and whether they can approach the concentrations measured by the commercially available ELISAs. It could also be tested how other amounts of immobilized aptamer or antibody and different ratios of capture, target, and reporter molecules might affect or even improve the assays. Furthermore, it would be interesting to test the assay on samples from cancer patients, as we have shown that the assay also works with human urine. In the case of human urine, samples could be pretreated, e.g., by centrifugation or filtration, to eliminate interfering factors (e.g., cells) and to obtain an even better detection limit. Urine samples can also be pretreated by concentrating the analyte to ensure better detection, as uPA is generally present at lower concentrations.

The use of the aptamers in electrochemical aptamer-based sensors [33,34] or lateral flow devices [35,36,37]—for cost-effective point-of-care testing (POCT)—could also be considered, as they also provide a high signal-to-noise ratio (low background signal), which usually improves sensitivity [27].

## 5. Conclusions

In conclusion, we successfully developed aptamer-based sandwich assay systems for the rapid detection of human HMW- and LMW-uPA with high specificity and sensitivity. The different sandwich assay formats are: 1. aptamer–target–aptamer, 2. aptamer–target–antibody, and 3. antibody–target–aptamer. The best combinations for detecting HMW-uPA were uPAapt−08−FR as the capture molecule and uPAapt−02−FR as the reporter molecule (format 1), uPAapt−02−FR as the capture molecule and Antibody Chain B as the reporter molecule (format 2), and Antibody Chain A as capture molecule and uPAapt− 02−FR as reporter molecule (format 3). The best combinations for detecting LMW-uPA was uPAapt−02−R as capture molecule and Antibody Chain B as reporter molecule (format 2) or Antibody Chain B as capture molecule and uPAapt−02 as reporter molecule (format 3). Since uPA is a highly discussed biomarker for several types of cancer, this aptamer-based assay can be used as a cost-effective alternative to commercially available antibody-based ELISAs.

## 6. Patents

All aptamers used in this manuscript are protected by pending patents.

## Data Availability

The datasets for the current study are available from the corresponding author on request.

## Supplementary Materials

The following supporting information can be downloaded at: https://www.mdpi.com/article/10.3390/cancers14215222/s1, Figure S1: Binding of the exchange sequences (Ex−S1–Ex−S42) to HMW-uPA by FLAA.; Table S1: Exchange sequences of uPAapt−02−FR.

## References

[1] Zou J., Wang E. (2019). Cancer biomarker discovery for precision medicine: New progress. Curr. Med. Chem..

[2] Su S.-C., Lin C.-W., Yang W.-E., Fan W.-L., Yang S.-F. (2016). The urokinase-type plasminogen activator (uPA) system as a biomarker and therapeutic target in human malignancies. Expert Opin. Ther. Targets.

[3] Mahmood N., Mihalcioiu C., Rabbani S.A. (2018). Multifaceted role of the urokinase-type plasminogen activator (uPA) and its receptor (uPAR): Diagnostic, prognostic, and therapeutic applications. Front. Oncol..

[4] Mekkawy A.H., Pourgholami M.H., Morris D.L. (2014). Involvement of urokinase-type plasminogen activator system in cancer: An overview. Med. Res. Rev..

[5] Dass K., Ahmad A., Azmi A.S., Sarkar S.H., Sarkar F.H. (2008). Evolving role of uPA/uPAR system in human cancers. Cancer Treat. Rev..

[6] Foekens J.A., Peters H.A., Look M.P., Portengen H., Schmitt M., Kramer M.D., Brünner N., Jänicke F., Meijer-van Gelder M.E., Henzen-Logmans S.C. (2000). The urokinase system of plasminogen activation and prognosis in 2780 breast cancer patients. Cancer Res..

[7] Duffy M.J., McGowan P.M., Harbeck N., Thomssen C., Schmitt M. (2014). uPA and PAI-1 as biomarkers in breast cancer: Validated for clinical use in level-of-evidence-1 studies. Breast Cancer Res..

[8] Harris L., Fritsche H., Mennel R., Norton L., Ravdin P., Taube S., Somerfield M.R., Hayes D.F., Bast R.C. (2007). American society of clinical oncology 2007 update of recommendations for the use of tumor markers in breast cancer. J. Clin. Oncol..

[9] Banys-Paluchowski M., Witzel I., Aktas B., Fasching P.A., Hartkopf A., Janni W., Kasimir-Bauer S., Pantel K., Schön G., Rack B. (2019). The prognostic relevance of urokinase-type plasminogen activator (uPA) in the blood of patients with metastatic breast cancer. Sci. Rep..

[10] Shariat S.F., Roehrborn C.G., McConnell J.D., Park S., Alam N., Wheeler T.M., Slawin K.M. (2007). Association of the circulating levels of the urokinase system of plasminogen activation with the presence of prostate cancer and invasion, progression, and metastasis. J. Clin. Oncol..

[11] Shariat S.F., Semjonow A., Lilja H., Savage C., Vickers A.J., Bjartell A. (2011). Tumor markers in prostate cancer I: Blood-based markers. Acta Oncol..

[12] Yang J.-L., Seetoo D.-q., Wang Y., Ranson M., Berney C.R., Ham J.M., Russell P.J., Crowe P.J. (2000). Urokinase-type plasminogen activator and its receptor in colorectal cancer: Independent prognostic factors of metastasis and cancer-specific survival and potential therapeutic targets. Int. J. Cancer.

[13] Herszényi L., Farinati F., Cardin R., István G., Molnár L.D., Hritz I., de Paoli M., Plebani M., Tulassay Z. (2008). Tumor marker utility and prognostic relevance of cathepsin B, cathepsin L, urokinase-type plasminogen activator, plasminogen activator inhibitor type-1, CEA and CA 19-9 in colorectal cancer. BMC Cancer.

[14] Shariat S.F., Monoski M.A., Andrews B., Wheeler T.M., Lerner S.P., Slawin K.M. (2003). Association of plasma urokinase-type plasminogen activator and its receptor with clinical outcome in patients undergoing radical cystectomy for transitional cell carcinoma of the bladder. Urology.

[15] Schuettfort V.M., Pradere B., D’Andrea D., Grossmann N.C., Quhal F., Mostafaei H., Laukhtina E., Mori K., Rink M., Karakiewicz P.I. (2021). Prognostic impact of preoperative plasma levels of urokinase plasminogen activator proteins on disease outcomes after radical cystectomy. J. Urol..

[16] Casella R., Shariat S.F., Monoski M.A., Lerner S.P. (2002). Urinary levels of urokinase-type plasminogen activator and its receptor in the detection of bladder carcinoma. Cancer.

[17] Shariat S.F., Casella R., Monoski M.A., Sulser T., Gasser T.C., Lerner S.P. (2003). The addition of urinary urokinase-type plasminogen activator to urinary nuclear matrix protein 22 and cytology improves the detection of bladder cancer. J. Urol..

[18] Sabrowski W., Dreymann N., Möller A., Czepluch D., Albani P.P., Theodoridis D., Menger M.M. (2022). The use of high-affinity polyhistidine binders as masking probes for the selection of an NDM-1 specific aptamer. Sci. Rep..

[19] Toh S.Y., Citartan M., Gopinath S.C.B., Tang T.-H. (2015). Aptamers as a replacement for antibodies in enzyme-linked immunosorbent assay. Biosens. Bioelectron..

[20] Bakhtiari H., Palizban A.A., Khanahmad H., Mofid M.R. (2020). Aptamer-based approaches for in vitro molecular detection of cancer. Res. Pharm. Sci..

[21] Dreymann N., Wuensche J., Sabrowski W., Moeller A., Czepluch D., Vu Van D., Fuessel S., Menger M.M. (2022). Inhibition of human urokinase-type plasminogen activator (uPA) enzyme activity and receptor binding by DNA aptamers as potential therapeutics through binding to the different forms of uPA. IJMS.

[22] Shrivastava A., Gupta V. (2011). Methods for the determination of limit of detection and limit of quantitation of the analytical methods. Chron. Young Sci..

[23] Zuker M. (2003). Mfold web server for nucleic acid folding and hybridization prediction. Nucleic Acids Res..

[24] Duffy M.J., Duggan C., Mulcahy H.E., McDermott E.W., O’Higgins N.J. (1998). Urokinase plasminogen activator: A prognostic marker in breast cancer including patients with axillary node-negative disease. Clin. Chem..

[25] Benraad T.J., Geurts-moespot J., Grøndahl-hansen J., Schmitt M., Heuvel J.J.T.M., de Witte J.H., Foekens J.A., Leake R.E., Brünner N., Sweep C.G.J. (1996). Immunoassays (ELISA) of urokinase-type plasminogen activator (uPA): Report of an EORTC/BIOMED-1 Workshop. Eur. J. Cancer.

[26] Luzi E., Minunni M., Tombelli S., Mascini M. (2003). New trends in affinity sensing. TrAC Trends Anal. Chem..

[27] Kalra P., Dhiman A., Cho W.C., Bruno J.G., Sharma T.K. (2018). Simple methods and rational design for enhancing aptamer sensitivity and specificity. Front. Mol. Biosci..

[28] Abeydeera N.D., Egli M., Cox N., Mercier K., Conde J.N., Pallan P.S., Mizurini D.M., Sierant M., Hibti F.-E., Hassell T. (2016). Evoking picomolar binding in RNA by a single phosphorodithioate linkage. Nucleic Acids Res..

[29] Lee K.Y., Kang H., Ryu S.H., Lee D.S., Lee J.H., Kim S. (2010). Bioimaging of nucleolin aptamer-containing 5-(*N*-benzylcarboxyamide)-2′-deoxyuridine more capable of specific binding to targets in cancer cells. J. Biomed. Biotechnol..

[30] Lipi F., Chen S., Chakravarthy M., Rakesh S., Veedu R.N. (2016). In vitro evolution of chemically-modified nucleic acid aptamers: Pros and cons, and comprehensive selection strategies. RNA Biol..

[31] Gold L., Ayers D., Bertino J., Bock C., Bock A., Brody E.N., Carter J., Dalby A.B., Eaton B.E., Fitzwater T. (2010). Aptamer-based multiplexed proteomic technology for biomarker discovery. PLoS ONE.

[32] Hernandez F.J., Kalra N., Wengel J., Vester B. (2009). Aptamers as a model for functional evaluation of LNA and 2′-amino LNA. Bioorg. Med. Chem. Lett..

[33] Yang Y., Yang X., Yang Y., Yuan Q. (2018). Aptamer-functionalized carbon nanomaterials electrochemical sensors for detecting cancer relevant biomolecules. Carbon.

[34] Bahner N., Reich P., Frense D., Menger M., Schieke K., Beckmann D. (2018). An aptamer-based biosensor for detection of doxorubicin by electrochemical impedance spectroscopy. Anal. Bioanal. Chem..

[35] Huang L., Tian S., Zhao W., Liu K., Ma X., Guo J. (2021). Aptamer-based lateral flow assay on-site biosensors. Biosens. Bioelectron..

[36] Dalirirad S., Steckl A.J. (2020). Lateral flow assay using aptamer-based sensing for on-site detection of dopamine in urine. Anal. Biochem..

[37] Frohnmeyer E., Tuschel N., Sitz T., Hermann C., Dahl G.T., Schulz F., Baeumner A.J., Fischer M. (2019). Aptamer lateral flow assays for rapid and sensitive detection of cholera toxin. Analyst.

