# Supplementary material for: Aptamer-Based Sandwich Assay Formats for Detection and Discrimination of Human High- and Low-Molecular-Weight uPA for Cancer Prognosis and Diagnosis
Source: Cancers (Basel). 2022 Oct 25;14(21):5222. doi: 10.3390/cancers14215222 (PMC9658990; doi:10.3390/cancers14215222)
Supplement: Supplementary file 1 [file cancers-14-05222-s001.zip › cancers-1956676-supplementary.pdf]

Supplementary Materials: Aptamer-based Sandwich Assay Formats for Detection and Discrimination of High- and Low-Molecular-Weight uPA for Cancer Prognosis and Diagnosis

Nico Dreymann [1,2], Wiebke Sabrowski [1,3], Jennifer Danso [1,4] and Marcus M. Menger [1,*]

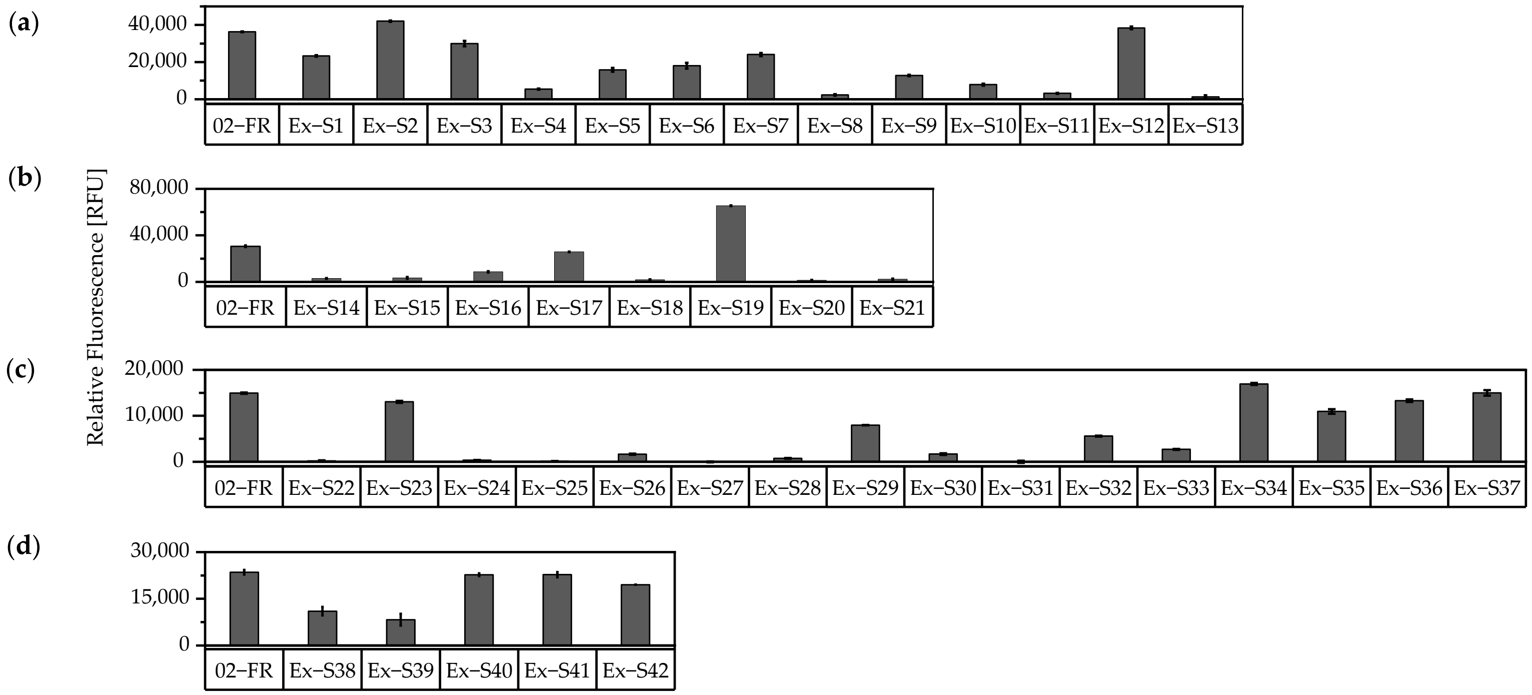

Figure S1. Binding of the exchange sequences (Ex−S1 – Ex−S42) to HMW-uPA by FLAA. Binding experiments were performed on different micro titer plates and are therefore shown as individual diagrams with the respective positive control uPAapt−02−FR (02−FR). (**a**) Ex−S1 – Ex−S13 (**b**) Ex−S14 – Ex−S21 (**c**) Ex−S22 – Ex−S37 (**d**) Ex−S38 – Ex−S42. The relative fluorescence unit [RFU] for each sample is given as the mean value of technical replicates and was measured using the multimode microplate reader Mithras² LB943. Negative control values were subtracted. Error bars represent the range of measured values of signals from the two target-coated wells plus the range of the measured values of the two negative control wells. Number of records *n=2.*

Table S1. Exchange sequences of uPAapt−02−FR. Each nucleotide of the sequence of uPAapt−02−FR was replaced individually (for G/C a T and for A/T a C). All 42 new sequence aptamers (Ex−S1 – Ex−S42) were tested for binding to HMW-uPA by FLAA. The reduction in fluorescence signal compared to the control uPAapt−02−FR is given as a percentage for each sequence. Exchanged nucleotides in each sequence are shown in red.

Name	Sequence	Signal reduction compared to control (uPAapt−02−FR)
Ex−S1	TAAGCGGGGGTGAGAGATCTGTCAGTACGAGCTGGGTTTGCG	36%
Ex−S2	CCAGCGGGGGTGAGAGATCTGTCAGTACGAGCTGGGTTTGCG	n.r.*
Ex−S3	CACGCGGGGGTGAGAGATCTGTCAGTACGAGCTGGGTTTGCG	18%
Ex−S4	CAATCGGGGGTGAGAGATCTGTCAGTACGAGCTGGGTTTGCG	85%
Ex−S5	CAAGTGGGGGTGAGAGATCTGTCAGTACGAGCTGGGTTTGCG	56%
Ex−S6	CAAGCTGGGGTGAGAGATCTGTCAGTACGAGCTGGGTTTGCG	50%
Ex−S7	CAAGCGTGGGTGAGAGATCTGTCAGTACGAGCTGGGTTTGCG	34%
Ex−S8	CAAGCGGTGGTGAGAGATCTGTCAGTACGAGCTGGGTTTGCG	94%
Ex−S9	CAAGCGGGTGTGAGAGATCTGTCAGTACGAGCTGGGTTTGCG	65%
Ex−S10	CAAGCGGGGTTGAGAGATCTGTCAGTACGAGCTGGGTTTGCG	78%
Ex−S11	CAAGCGGGGGCGAGAGATCTGTCAGTACGAGCTGGGTTTGCG	91%
Ex−S12	CAAGCGGGGGTTAGAGATCTGTCAGTACGAGCTGGGTTTGCG	n.r.*
Ex−S13	CAAGCGGGGGTGCGAGATCTGTCAGTACGAGCTGGGTTTGCG	97%
Ex−S14	CAAGCGGGGGTGATAGATCTGTCAGTACGAGCTGGGTTTGCG	91%
Ex−S15	CAAGCGGGGGTGAGCGATCTGTCAGTACGAGCTGGGTTTGCG	89%
Ex−S16	CAAGCGGGGGTGAGATATCTGTCAGTACGAGCTGGGTTTGCG	72%
Ex−S17	CAAGCGGGGGTGAGAGCTCTGTCAGTACGAGCTGGGTTTGCG	16%
Ex−S18	CAAGCGGGGGTGAGAGACCTGTCAGTACGAGCTGGGTTTGCG	94%
Ex−S19	CAAGCGGGGGTGAGAGATTTGTCAGTACGAGCTGGGTTTGCG	0%*
Ex−S20	CAAGCGGGGGTGAGAGATCCGTCAGTACGAGCTGGGTTTGCG	95%
Ex−S21	CAAGCGGGGGTGAGAGATCTTTCAGTACGAGCTGGGTTTGCG	92%
Ex−S22	CAAGCGGGGGTGAGAGATCTGCCAGTACGAGCTGGGTTTGCG	99%
Ex−S23	CAAGCGGGGGTGAGAGATCTGTTAGTACGAGCTGGGTTTGCG	13%
Ex−S24	CAAGCGGGGGTGAGAGATCTGTCCGTACGAGCTGGGTTTGCG	98%
Ex−S25	CAAGCGGGGGTGAGAGATCTGTCATTACGAGCTGGGTTTGCG	99%
Ex−S26	CAAGCGGGGGTGAGAGATCTGTCAGCACGAGCTGGGTTTGCG	89%
Ex−S27	CAAGCGGGGGTGAGAGATCTGTCAGTCCGAGCTGGGTTTGCG	100%
Ex−S28	CAAGCGGGGGTGAGAGATCTGTCAGTATGAGCTGGGTTTGCG	95%
Ex−S29	CAAGCGGGGGTGAGAGATCTGTCAGTACTAGCTGGGTTTGCG	47%
Ex−S30	CAAGCGGGGGTGAGAGATCTGTCAGTACGCGCTGGGTTTGCG	89%
Ex−S31	CAAGCGGGGGTGAGAGATCTGTCAGTACGATCTGGGTTTGCG	100%
Ex−S32	CAAGCGGGGGTGAGAGATCTGTCAGTACGAGTTGGGTTTGCG	63%
Ex−S33	CAAGCGGGGGTGAGAGATCTGTCAGTACGAGCCGGGTTTGCG	82%
Ex−S34	CAAGCGGGGGTGAGAGATCTGTCAGTACGAGCTTGGTTTGCG	0%*
Ex−S35	CAAGCGGGGGTGAGAGATCTGTCAGTACGAGCTGTGTTTGCG	27%
Ex−S36	CAAGCGGGGGTGAGAGATCTGTCAGTACGAGCTGGTTTTGCG	11%
Ex−S37	CAAGCGGGGGTGAGAGATCTGTCAGTACGAGCTGGGCTTGCG	n.r.*
Ex−S38	CAAGCGGGGGTGAGAGATCTGTCAGTACGAGCTGGGTCTGCG	53%
Ex−S39	CAAGCGGGGGTGAGAGATCTGTCAGTACGAGCTGGGTTCGCG	65%
Ex−S40	CAAGCGGGGGTGAGAGATCTGTCAGTACGAGCTGGGTTTTCG	3%
Ex−S41	CAAGCGGGGGTGAGAGATCTGTCAGTACGAGCTGGGTTTGTG	3%
Ex−S42	CAAGCGGGGGTGAGAGATCTGTCAGTACGAGCTGGGTTTGCT	17%

Note: n.r.*, no reduction (equal or higher signal compared to control uPAapt−02−FR).
